# Unit Load of Abrasive Grains in the Machining Zone During Microfinishing with Abrasive Films

**DOI:** 10.3390/ma17246305

**Published:** 2024-12-23

**Authors:** Katarzyna Tandecka, Wojciech Kacalak, Filip Szafraniec, Thomas G. Mathia

**Affiliations:** 1 Department of Engineering and Informatics Systems, Faculty of Mechanical Engineering and Energy, Koszalin University of Technology, 75-620 Koszalin, Poland; wojciech.kacalak@tu.koszalin.pl (W.K.); filip.szafraniec@tu.koszalin.pl (F.S.); 2 Laboratoire de Tribologie et Dynamique des Systemes (LTDS), Ecole Centrale de Lyon, Centre National de la Recherche Scientifique, 69134 Lyon, France

**Keywords:** surface finishing, abrasive film, finishing, abrasion, superfinishing, unit load, abrasive grains

## Abstract

This work investigates the contact between abrasive particles and workpieces in microfinishing processes with special consideration given to the determination of unit force, unit pressure, and grain, the forces exerted by individual abrasive grains. A detailed methodology was established for measuring the contact area, penetration depth, and circumferences of grain imprints at depths corresponding to multiples of the total height of the abrasive film, represented by the parameter *Sz*. The following depths were analyzed: 0.05 *Sz*, 0.15 *Sz*, 0.25 *Sz*, and 0.35 *Sz*. Results show that the areas closer to the central microfinishing zone bear the highest unit pressures and forces and, thus, contribute dominantly to material removal. It was further found that near the edges of the contact zone, the pressure and force have been reduced to lower material removal efficiency. The non-uniform geometry of abrasive particles was found to significantly affect contact mechanics, more at shallow depths of penetration, whereas the shape of the apex defines the nature of the interaction. A parabolic force and pressure distribution were evident for the irregular load distribution of the microfinishing area. The result brings out the need for further refinement in the design of the abrasive film and pressure distribution in order to achieve improvement in uniformity and efficiency during microfinishing. It would bring out valuable insights on how to improve the effectiveness of an abrasive film and ways of optimizing the process conditions. The results provide a founding stone for further advancement of knowledge in the grain–workpiece interaction, enabling better surface quality and more reliable microfinishing processes.

## 1. Introduction

Microfinishing is one of the most important operations in obtaining good surface finishes, mainly in such industries as aerospace, automotive, and optical. It is based on the interaction between abrasive particles and a workpiece, allowing the removal of material at the microscale and improving the characteristics of a surface under strict tolerances. Of the myriad abrasive tools, microfinishing films have gained prominence due to their controlled grain distribution, flexibility, and ability to produce consistent results over a wide range of pressure conditions. Despite being widely used, mechanisms governing grain–workpiece interaction, including force distribution, material removal efficiency, and the role of grain geometry, are still under active research. A deeper understanding of these mechanisms is crucial for improving the predictability and efficiency of microfinishing processes, particularly for advanced materials and applications requiring high surface quality. However, many existing studies fail to address key challenges encountered in industrial applications. For instance, the scalability of laboratory findings to high-volume manufacturing remains problematic, particularly when dealing with variable material properties. Moreover, sustainability considerations, such as extending abrasive tool life and minimizing waste, are often neglected. Abrasives used in microfinishing processes are normally single-use; hence, it is of high importance to make maximum utilization of the abrasive surface while the process is going on for better efficiency and economy without wastage of scarce resources. Addressing these gaps will bridge the divide between theoretical findings and practical industrial needs toward efficient ecological responsibility in microfinishing processes.

**Figure 1:**
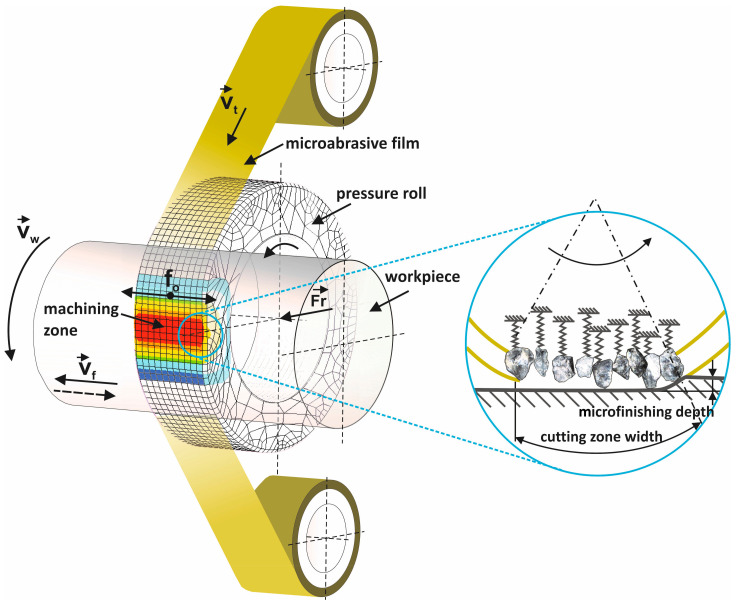
A kinematic diagram illustrating rotary surface finishing with lapping films, highlighting the following parameters within the diagram: *v_t_*—tool speed, *v_w_*—workpiece speed, *v_f_*—tool feed speed, *f_o_*—tool oscillation frequency, and *F_r_*—the pressure force of the pressing roller [10].

Microfinishing by using abrasive films is one of the precision surface finishing methods to achieve high-quality finishes with minimum material removal, as shown in Figure 1. In this process, a flexible microabrasive film is brought into controlled contact with the workpiece surface. The workpiece rotates at a high speed, *v_w_*, whereas the abrasive film moves with a low speed, *vt*, during one pass along the machining zone so that the film contacts the work surface of the workpiece once [1]. This is a critical condition to ensure the film maintains its homogeneity without causing excessive wear of the workpiece. A controlled normal force, *Fr*, is exerted by the pressure roll, which presses the abrasive film against the rotating workpiece to create a defined machining zone. Inside this zone, abrasive grains on the film penetrate into the workpiece surface and, thus, enable material removal on a microscale [2,3]. The schematic inset illustrates in detail a drawing of the depth of microfinishing related to the width of the cutting zone and depicts the interaction of individual grains with the work surface. The springs shown in the inset represent elastic deformation and recovery of the grains during the cutting action, showing that the grains are active in involving themselves with the surface to achieve the desired finish [4,5]. That creates control to accurately remove the material removal process and, therefore, control the resultant surface quality [6]. To avoid over-processing and resultant heat generation, the engagement is limited to one pass, while the film movement is also kept low [7,8]. The combination of a high workpiece velocity with low film speed and also a low-pressure application makes the process of microfinishing using an abrasive film very precise and efficient to get fine surface finishes within narrow tolerances [9].

**Figure 2:**
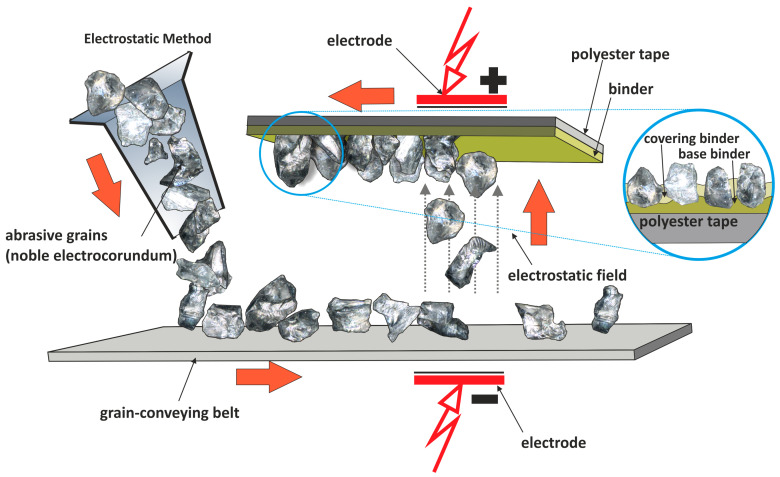
The manufacturing process scheme of microfinishing films within an electrostatic field [12].

The abrasive films used in microfinishing are prepared through a special process meant for the proper orientation of abrasive particles [11] (Figure 2). These particles, when deposited on the surface of the film, are evenly distributed in an electrostatic field and, hence, aligned properly. Thus, it holds the particles in position, spaced correctly to each particle with respect to the work surface for maximum efficiency in cutting and a fine, even finish obtained on the work. Application of the electrostatic field in the process allows for better control over the thickness and, more importantly, the density of the grain layer, which is very critical in obtaining good quality surfaces in microfinishing.

In microfinishing with coated abrasive tools, there is an intricate interaction between the workpiece surface and the tool elastically pressed within the machining zone [13,14,15]. Such interaction is mainly controlled by the mechanical properties of abrasive grains, elasticity in the abrasive film backing, applied force, and the workpiece material characteristics. The machining zone becomes a dynamic system in which different physical phenomena influence simultaneously the material removal process, the surface integrity, and the tool efficiency [16,17,18]. When the abrasive tool is pressed onto the workpiece, grains embedded in the flexible film make the first contact with the surface [19,20]. Inasmuch as the backing is elastic, not all grains are equally in contact with the workpiece at any given instant; only those grains of the highest protrusions are engaging in material removal [2,21,22]. Selective contact is further altered by an applied normal force, compressing the abrasive film and increasing the engaged grains as force is applied. The elastic backing ensures that the abrasive tool conforms to surface irregularities on the workpiece, thereby improving the contact and uniform pressure distribution over the machining area [23,24,25].

In general, material removal in the machining zone is caused by two mechanisms: micro-cutting and plowing. As the force is applied, abrasive particles penetrate the workpiece surface; then, sharper grains tend to act as a micro-cutting tool, removing material in tiny chips [26]. At the same time, some particles, especially those with dull edges or low penetration, plow and compress the surface by causing plastic deformation [27,28,29]. In this way, the double action of these particles leads to the removal of material and, simultaneously, the improvement of the surface quality, which will finally become smooth and glossy [3,30,31]. The shape of the abrasive grains is important for determining the nature of their contact with the workpiece [32,33,34]. Those grains having an irregular shape provide a more complex contact profile and thereby increase the contact points, hence raising the material removal rate. Upon being elastically deformed, it adapts itself to grains of varied height and shape and consequently provides a more homogeneous distribution of force. This, however, implies that the grains closer to the center of the machining zone, where the force applied is maximum, will penetrate deeper and will have a greater contribution to surface finish than those grains at the peripheral regions.

This friction between the workpiece and the abrasive particles produces thermal energy in the machining zone. The flexible substrate and single-pass configuration of the abrasive films tend to reduce the excessive heat generation; however, there could still be some localized temperature increases around each grain due to the high pressure and friction [35,36,37]. Those thermal variations may lead to minor thermal effects in the workpiece material by way of softening or thermal expansion, thus affecting the machining dynamics. During material removal, it forms chips and debris that have to be effectively cleared from the machining zone for effective tool–workpiece interaction [38,39,40]. This flexibility of the abrasive film allows the spaces between grains to act as chip storage reservoirs, avoiding clogging and hence retaining the cutting efficiency [41,42]. Another contribution to the self-cleaning mechanism of the film is an elastic deformation of the backing by flexing and releasing trapped debris while it operates [33,43].

The elastic backing creates a non-uniform force distribution in the machining zone. In general, the forces are higher in the central regions and lower near the edges. This gradient gives a parabolic pressure distribution as has been experimentally found. The primary regions, which have high force and pressure, support the major part of the load during the microfinishing operation and, thus, enable the highest material removal [44,45,46]. On the other hand, lower interaction intensities are expected in the peripheral regions, where the pressures are low, resulting in less effectiveness of material removal. The complex dynamics of the phenomena involved underline the importance of understanding contact mechanics, abrasive grain geometry, and pressure distribution in the machining zone. Knowledge of this nature is very important for the optimization of microfinishing processes in order to achieve consistent surface finishes, raise the efficiency of material removal, and ensure abrasive film longevity.

The effectiveness of microfinishing primarily depends on contact mechanics between the abrasive grains and the workpiece. Parameters of the contact area, the depth of penetration, and grain surface features will be influential in both the rate of material removal and in achieving uniformity within a specified surface finish. Material removal studies in preceding times have indicated the prominence of the central contact zones under the action of the greatest pressure and forces to effect material removal. However, most peripheral areas show poor performance due to lower pressure amplitudes and less efficient grain interaction and hence need further study and enhancement.

Another important consideration is that the non-homogeneous geometry of the abrasive particles causes a serious effect on contact mechanics during shallow penetration depths. That is, the complex geometries of these grains induce variations in contact area and load distribution, which profoundly influences the overall effectiveness of the microfinishing process. These interactions are at least partly apprehended via a detailed quantitative analysis of parameters such as unit force, unit pressure, and forces acting on individual grains besides the circumferential development of grain imprints. This work is dedicated to the investigation of interaction dynamics between the abrasive grains and the workpiece in microfinishing with films, detailing the spatial distribution of contact areas, penetration depths, and forces within predetermined contact zones. A new method was developed for the measurement of the extent of surface development in grain imprints in order to investigate the parabolic force and pressure distribution patterns. The present interest in microfinishing films is driven by the fact that they are increasingly used in many industrial settings; with a better understanding of grain–workpiece interactions, further optimization of their performance is possible. The results of this research give the basic information necessary for abrasive film design and parameter optimization in microfinishing and, consequently, for improved efficiency and reliability in surface finishing operations.

## 2. Materials and Methods

### 2.1. Examination of Abrasive Film Surfaces

Topographical characteristics of microfinishing films were measured by an Olympus OLS4000 confocal microscope, Tokyo, Japan. The films to analyze were selected according to nominal grain sizes of 15 μm (15MFF). Measurement area sizes were 638 × 638 and 126 × 126 μm. The analyzed surface had 1024 × 1024 data points. Furthermore, observational experiments were carried out by objective lenses with 20× and 100× magnifications. A Phenom ProX tabletop scanning electron microscope (SEM) was used in order to examine the microfinishing film surfaces. Detailed observations of the abrasive surface were obtained using this equipment from Phenom-World BV, Eindhoven, The Netherlands.

**Figure 3:**
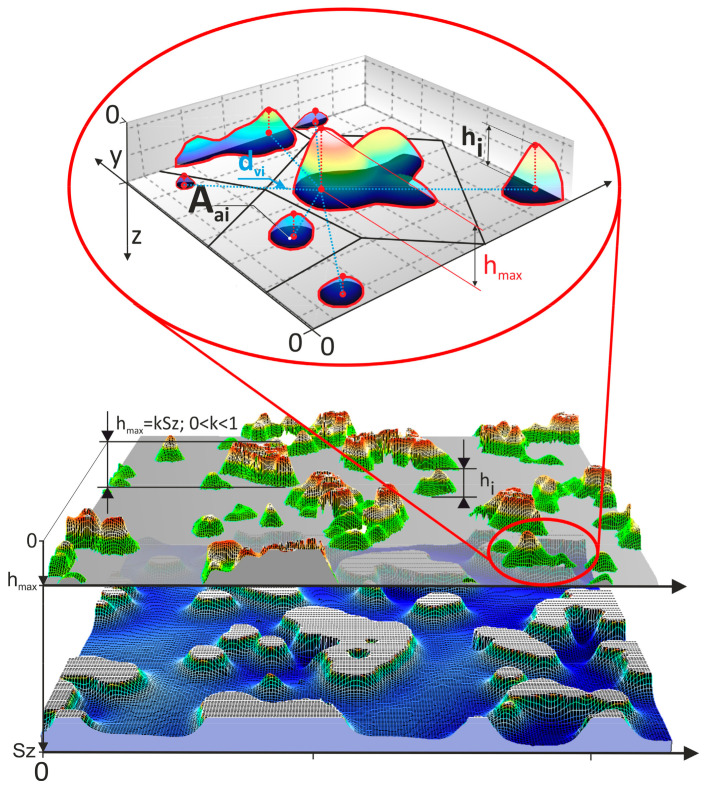
Methodology for surface analysis of abrasive films [47].

The methodology (Figure 3) for evaluating the surface of abrasive films, including active apexes, was proposed and described by the authors in this article [47].

The first screening of the machining capabilities of the microfinishing films was carried out through the estimation of peaks that would participate substantially in the microfinishing process [30,48,49]. To this end, a method known as peak truncation was employed. First of all, the calculation of maximum surface height, also called *Sz* (maximum height of the surface), was carried out. The vertical distance was measured from the highest elevation of an abrasive grain to the lowest elevation at the binder level on the surface of the abrasive film. In the next step, the truncation plane was defined separating the peaks of grains from the rest of the tool structure. A plane was placed at a height, referred to as *h_max_* here, between the highest surface peak (Figure 3). The maximum value of *h*, referred to as hereinafter by using actual values 0.05 *Sz*, 0.15 *Sz*, 0.25 *Sz*, and 0.35 *Sz* herein the coefficient *k* to determine may be expressed in terms of a product expression from *Sz*.

Previous work [10] has shown that in machining, abrasive grains penetrate into the work material to a depth of about 10 percent of their nominal size. Now, taking into consideration the abovementioned fact and the circumstance that the abrasive grains are unevenly distributed along the abrasive sheet, sometimes, it might happen that abrasive clusters in localized areas are formed. In this regard, classically, it is assumed that those particles, which lie deeper than 0.35 *Sz* from the surface, do not take part any longer in the machining process. In this way, that research area was defined for a preliminary investigation. In another sense, these surfaces behave as containers for the waste of the machining operation, like chips of the work material [12]. The peaks were removed from the surface and taken for a closer examination in order to see other remaining peaks of the abrasive film. The number of those peaks was measured, and their heights, denoted as *hi*, were evaluated (for *i* = 1 to *n*). The highest peak in the grains studied was used as a reference point to calculate distances *d_vi_* between neighboring grains. The latter was the average of the distances between one grain and its nearest neighbors, which were determined by the Voronoi cell method. To obtain the projected area of each individual grain, *A_ai_*, integration was performed over each of the peaks (for *i* = 1 to *n*), as illustrated in Figure 3.

**Figure 4:**
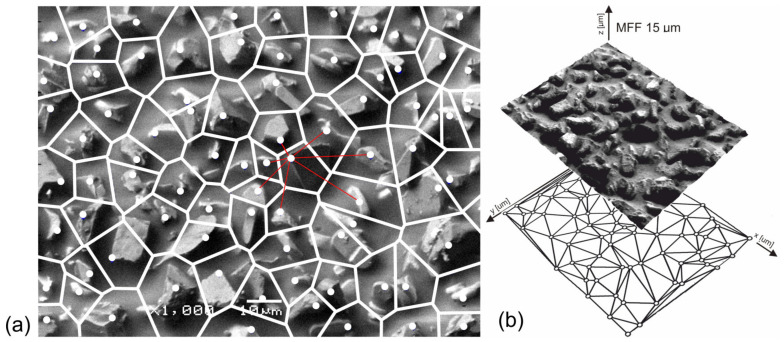
Division of the abrasive film surface into Voronoi cells (**a**) and projection of distances determined between the film vertices using the Voronoi cell method (**b**) [47].

This study will now calculate the distances between grains by marking the nearest neighbors of each grain peak by dividing the surface into Voronoi cells (Figure 4a). The tip of every bump, representing the peak of a grain, has been used as the coordinate reference for its corresponding Voronoi cell, hence allowing deeper analyses regarding the distribution of grain spacing over the whole surface and understanding their pattern and density.

The Voronoi tessellation method, a common approach in the material science and image analysis communities to subdivide the surface into discrete regions based on geometric proximity, groups all points that are closer to its central grain peak than to any other peak into each cell. Nearest neighbors were determined for each grain by this method, and the average nearneighbor peak distance was calculated. This distribution of distances is represented graphically by the Voronoi diagram (Figure 4b). It should be noted that the heights of single grains of abrasives were not measured. Still, the SEM picture creates a possibility to imagine what might be the closest neighborhood of each grain seen in the picture. Under these conditions, the developed method presents the possibility of obtaining data related to the spatial distribution of abrasive grains, which is very relevant in explaining their behavior in the surface finishing process.

### 2.2. Analysis of the Interaction of Abrasive Grains with the Workpiece

**Figure 5:**
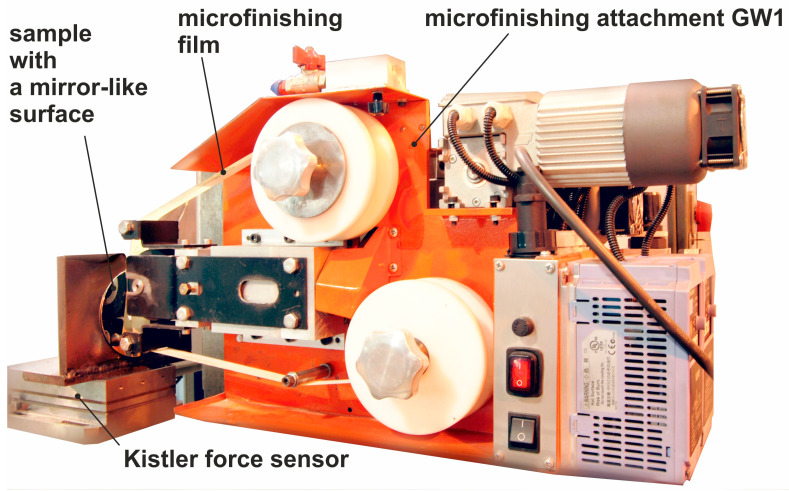
Test stand for analyzing the interaction of abrasive grains with the workpiece.

**Figure 6:**
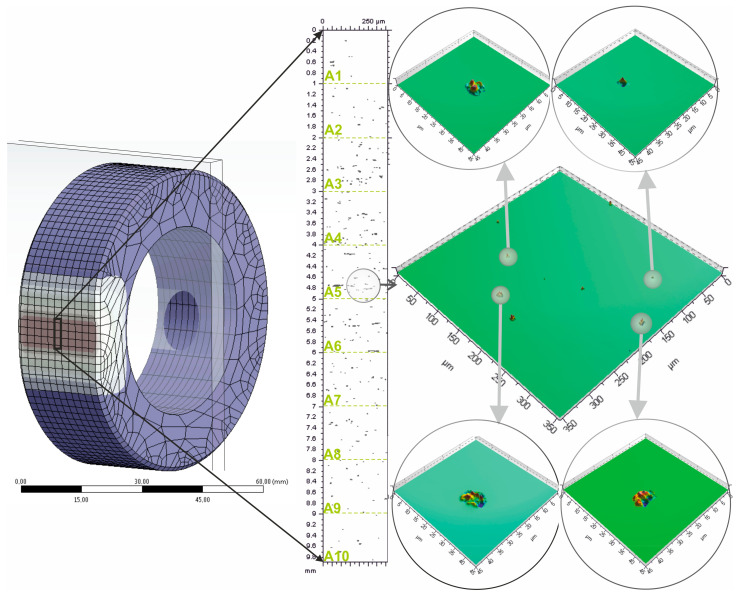
Image of the contact zone between the abrasive film and the workpiece, showing visible impressions of the grains on its surface.

In order to conduct a deep investigation on the possible action of abrasive particles, studies pertaining to the pressure application from an abrasive film on a specimen surface having a highly polished texture were carried out. The workpiece and abrasive film particles were studied on the GW–1 setup provided with the pressing roller of hardness 90 Shore A (Figure 5). The specimen was compressed by the roller through a force of 300 N, which gave a contact area of 10 mm in length (Figure 6). A so-called initial surface was taken, which should be as smooth and reflective as possible—on this occasion—to suppress the residual impressions coming from the previous treatments and within a maximum height restriction of 4.5 nanometers. Workpieces subjected in the present investigation were on amorphous nickel–phosphorus alloy. This material was chosen because it contains no crystalline structure that would not interfere with the observation of machining marks left on the surface during the interaction of abrasive grains; it was hard enough to permit effective evaluation of the microabrasive films. An essential part of the research was based on the force exerted by abrasive particles on the processed surface. In order to help in this investigation, a special experimental setup was designed (Figure 5). In summary, the pressing process conditions are as follows: a pressing roller with a hardness of 90° ShA, a pressing force of 300 N, a machining zone length of 10 mm, and the sample material is an amorphous NiP alloy. In the experiments, the GW-1 microfinishing accessory for external cylindrical surfaces was used. In the setup, an unobstructed pressing surface was provided to allow the contact area of the rollers with the flat surface to be measured accurately. A roller was fixed to the GW–1 attachment; it applied pneumatic pressure on the machined surface. The pressing mechanism is coupled with a piezoelectric force sensor model 9257B by KISTLER, Winterthur, Switzerland. The sensor was connected with a KISTLER multi-channel signal amplifier, model 5070A 12100, and connected to a personal computer installed with a measurement card, model 2855A4, made by KISTLER. The pressing roller, due to pneumatic pressure, exerted force on the tested sample, which was mounted on a frame directly connected to the force gauge. Force readings were recorded in real time in the MATLAB 2018a software environment, ensuring precise monitoring and analysis of the applied pressure during the experiment.

**Figure 7:**
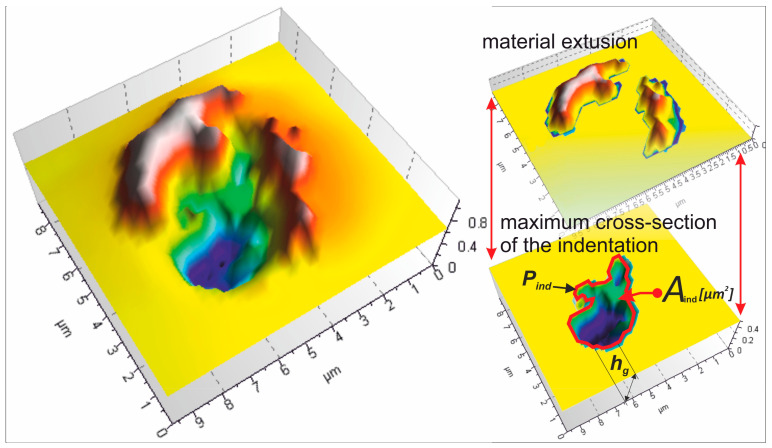
Methodology for analyzing the interaction traces of abrasive grain apexes on the machined surface.

The contact zone, 10 mm wide, was divided into ten subzones, A1...A10 (Figure 6), each 1 mm in width, starting from the beginning of the zone. Using a TalySurf CCI 6000 measurement system (Taylor Hobson, Leicester, England), the topography of the contact zone, including indentations resulting from the interaction of abrasive grains, was measured. The surface topography of the contact area is characterized by numerous indentations formed due to the pressure exerted by the abrasive film on the workpiece. For each indentation, the following features were determined: the depth of the indentation (*h_g_*), the maximum perimeter of the indentation (*P_ind_*), and the maximum cross-sectional area of the indentation (*A_ind_*) (Figure 7).

### 2.3. Methodology for Determining Unit Pressures Acting in the Contact Zone

The second step of interpretation consisted of calculating the unit force (*F_n,A_*), unit pressure (*q_A_*), and the normal force due to every single grain (*F_n,g_*) in every contact zone A1…A10. The methodological procedure was constructed in such a way as to determine the unit pressure (*q_A_*) as a function of the base area of the indentation (*A_ind_*) at the contact surface. From the above assumptions, the unit force (*F_n,A_*) is numerically equal to the unit pressure (*q_A_*), under the condition that the dimension of the investigated contact area (*A_z_*) is standardized to 1 mm^2^ (3). The relation between the unit force (*F_n,A_*) and the projected area of indentation (*A_ind_*) is described in terms of a function by Equation (1). In it, it acts as an origin for the relation of the force actually applied in the contact region to the resultant features measured on the single-grain imprint. Thereinto, Equation (2) has unit force (*F_n,A_*) as a function of coefficient (*C*) times summation of contact area, *A_sum_*, which depends on the accumulation of various isolated small contact areas *A_ind_* under a particular zone.

First of all, the single contact surface areas, *A_ind_*, for each indentation produced by abrasive grains were measured. The areas are a basis under the grain impressions and a basis for further calculations. Afterward, for each defined zone, the total contact area, *A_sum_*, was calculated by summation of all individual areas (2). To proceed further with the calculations, the coefficient *C* was calculated, which is defined as the ratio between the total force *F_T_* to the sum of the contact surface areas (*A_sum_*) across all zones, according to (5). This coefficient is required in the next steps for the determination of forces and pressures on the contact areas. Using the coefficient, *C*, and summation of contact area, *A_sum_*, unit force in each zone, *F_n,A_* was calculated by the following Equation (2). Since the contact area of the zone was normalized as 1 mm^2^, unit pressure, *q_A_*, was equal to unit force, *F_n,A_* (3). From the normal force of the assembling, *F_n,A_* was computed—by dividing by the actual number of grains in contact at that moment—the normal force acting on each single grain, *F_n,g_* (6). With this, a detailed look into the forces acting on the single grains during contact should be possible. It can be used in order to find out the way forces and pressures on the workpiece, supported in contact zones, might be established in the most precise way when interacting grains. Presented relations relating to total force with areas of contact and a number of grains make it possible to specify the justifiability and usefulness of used abrasive films during microfinishing operations.

(1) $$F_{n,A}=f\left(A_{ind}\right)$$

(2) $$F_{n,A}=C\cdot {\sum \;A_{ind}}^{k}=C\cdot A_{sum^{k}}$$

(3) $$q_{A}=F_{n,A}/A_{Z};A_{Z}=1mm^{2}$$

(4) $$F_{T}=\sum F_{n,A}=C\cdot \sum A_{sum^{k}}$$

(5) $$C=\frac{F_{T}}{\sum A_{sum^{k}}}$$

(6) $$F_{n,g}=\frac{F_{n,A}}{n_{i}}$$

*F_n,A_*—unit force in a zone with a surface area of 1 mm^2^, N;
*q_A_*—unit pressure in the contact zone, N/mm^2;^
*A_ind_*—contact surface area between the grain and the workpiece, µm^2;^
*F_T_*—total force, N;
*C*—coefficient of the ratio of total force to contact surface area;
*A_sum_*—sum of contact areas in the zone, µm^2;^
*F_n,g_*—force per single grain, N;
*n_i_*—number of grains in the zone.

## 3. Results and Discussion

### 3.1. Analysis of the Surface of Abrasive Films

**Figure 8:**
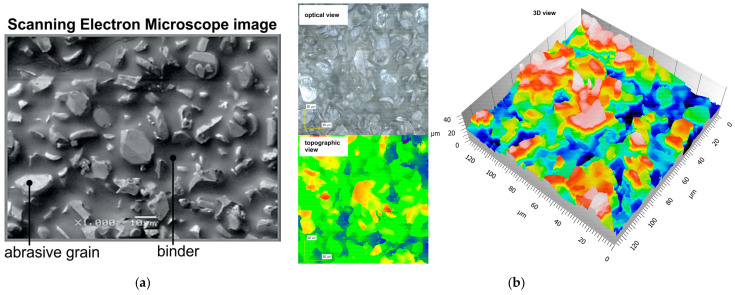
SEM image of the abrasive film surface with a nominal abrasive grain size of 15 μm (**a**) and results of topography analysis from a confocal microscope: optical view, topographic view, and 3D topographic view (**b**).

In the initial phase of the research, the topography of the surface was investigated with abrasive tools. In Figure 8a, the surface features are illustrated for an abrasive film with the binder layer coated by the abrasive particles embedded into it. It is a fact of great importance that these particles are not fully covered by the binder as they are positioned inside the binder in an electrostatic field. The nominal grain size of the studied abrasive film was 15 μm (15MFF). Confocal microscopy analysis results are presented in Figure 8b, where three different views are illustrated: an optical image, a 2D topographical map, and a 3D representation. The surface of the abrasive film is characterized by high variability in grain size, which also translates into differences in height for potentially cutting apexes.

**Figure 9:**
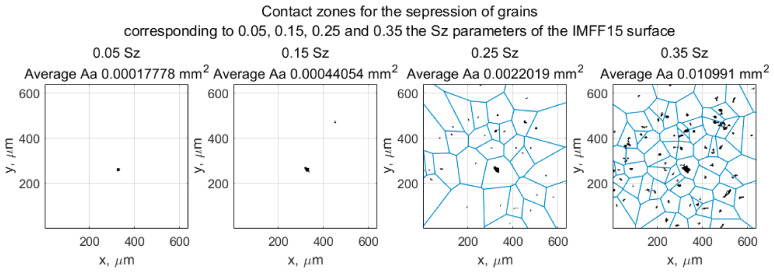
Division of the film surface into Voronoi cells, where the center is an active vertex depending on the position of the cutoff plane at successive levels: 0.05, 0.15, 0.25, and 0.35 *Sz* parameters [47].

The investigation focused on abrasive films, specifically the 15MFF type, to study their interaction with the workpiece. The cutting plane method was applied, where the plane was set at four different heights: 0.05, 0.15, 0.25, and 0.35 times the maximum height of surface irregularities, measured from the top of the surface of the film (Figure 9). In microfinishing processes under flexible pressure, only the peaks of abrasive grains take part in cutting; spaces between grains act as means for transporting machining by-products out of the processing zone. For the 15 μm abrasive film, when cutting at levels of 0.05 and 0.15 with regard to the maximum surface height, interactions between the tool and workpiece occurred occasionally with only one or two points of contact. This can be attributed to the random deposition of abrasive particles while manufacturing, even under the action of an electrostatic field aimed at orientation. In some cases, these particles overlap and form clusters, but this fact has negligible influence on machining performance. Due to the high unit pressure on a single particle upon touching it, it gets dislodged and, hence, increases the number of contact points as the cutting level increases—to 0.25, for instance. Voronoi cells, whose centers are located at the peaks of each grain, were used to study the nearest neighbor distances between cutting peaks. This approach can be used further to measure the free space available around each grain to store the machining by-products, which gives insight into the functional design of abrasive films like 15MFF during microfinishing.

**Figure 10:**
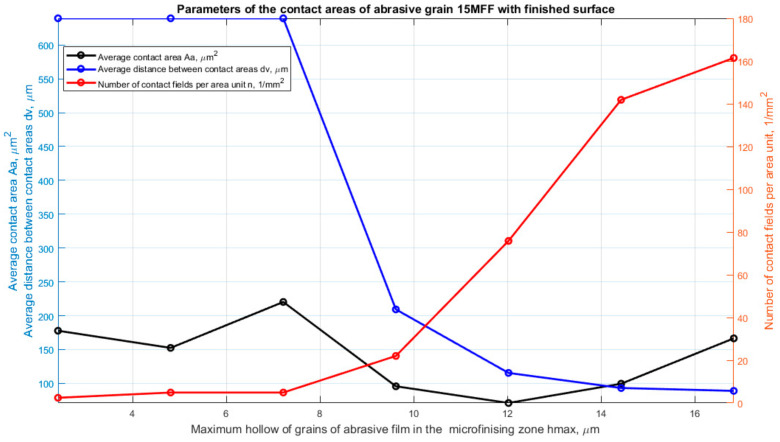
Parameters of potential contact between the abrasive film and the workpiece surface depending on the tool penetration depth into the material [47].

In the case of the 15MFF abrasive film, there were considered influential parameters concerning material removal effectiveness at different depths of penetration into the workpiece, ranging from 0.05 to 0.35 of the maximum height of surface irregularities. The contact area (Aa), spacing between contact points (dv), and total number of contact points (n) were analyzed. For these parameters, their average values for every single depth of penetration were calculated. The results shown in Figure 10 reveal that the maximum number of active peaks was measured at a penetration depth of 17 μm (0.35 *Sz*). At this particular depth, the inter-peak distance reached its minimum value, just below 200 μm. Additionally, it was found that at depths of up to about 7 μm beneath the cutting plane, the highest peaks, although comparatively sparse, would contribute most to the microfinishing process. Such high peaks correspond to larger grains, as confirmed by the parameter Aa, which showed a rise until this particular depth of penetration, after which it decreased and later increased again. However, during the investigated period, the parameter Aa did not return to its initial value at a depth of 7 μm. Similarly, the maximum number of peaks was recorded at the maximum penetration depth of 17 μm, thus justifying that more significant grains contribute significantly to the microfinishing process at this depth.

### 3.2. Study of the Forces Exerted by Abrasive Grains on the Workpiece

Before analyzing the contact areas between the tool and the workpiece, the surface topography of the sample was examined to eliminate any potential disturbances in the results caused by surface defects. After the surface analysis, the obtained image and parameters were determined according to the ISO 25178 standard [50] for surface characterization, specifically for the following height parameters:

Sp: maximum height of peaks;
Sv: maximum height of valleys;
*Sz*: maximum height of the surface;
Sa: arithmetical mean height of the surface.

**Figure 11:**
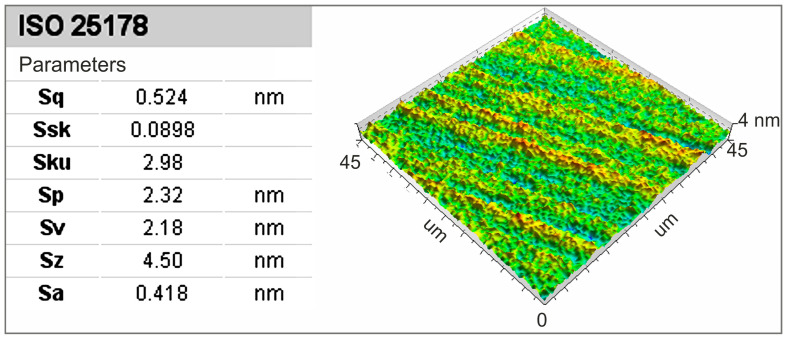
A very smooth sample surface for studying the interactions of abrasive grains with the machined surface, including determined parameters for surface roughness evaluation according to ISO 25178.

The results are presented in Figure 11. It was proven that the maximum surface irregularity height was 4.5 nm, while the nominal grain size was 15 μm. Even if a grain penetrates the workpiece material by 1 μm, such an irregularity would be immediately detectable due to the completely different order of magnitude of these values.

**Figure 12:**
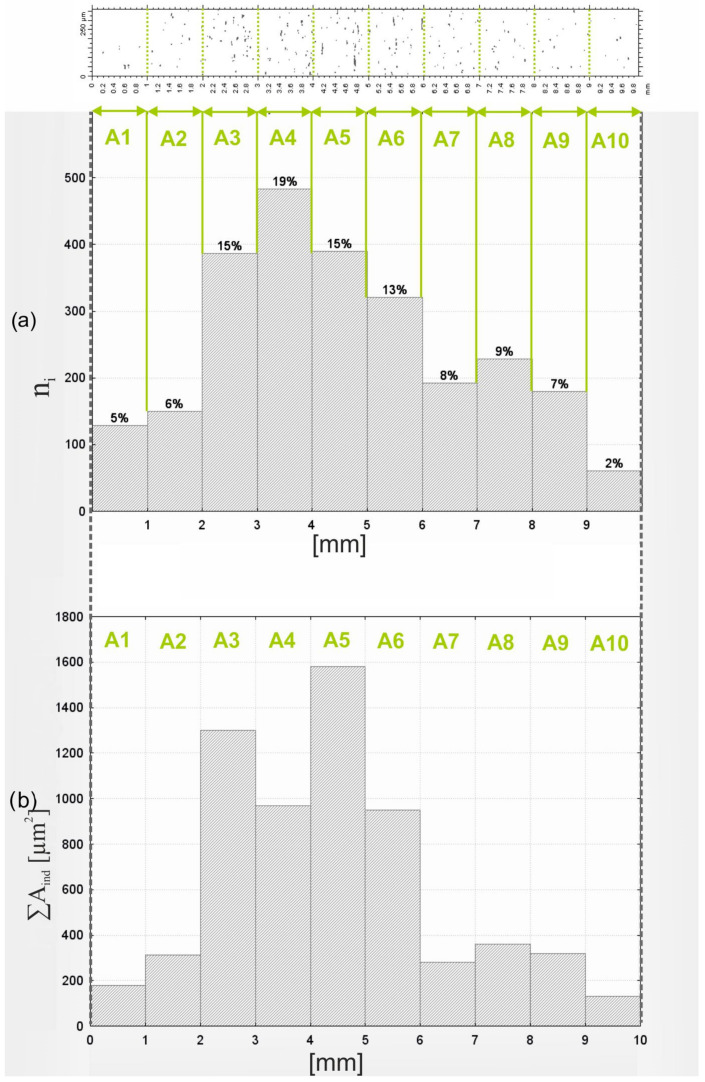
Histogram of the number of contacts along the entire length of the contact zone *n_i_* (**a**) and the total area of grain impressions in segments of the contact zone between the film and the workpiece *A_ind_* (**b**).

Figure 12 presents two histograms to analyze the characteristics of contact zones in the microfinishing process. The x-axis of both histograms indicates 10 subzones of the contact region, which are named A1 to A10, where the width of the subzone is 1 mm. Histogram (a) shows the number of abrasive grain contacts, so-called *n_i_*, covering the respective subzones, with percentage values above the bars indicating the distribution of these contacts over the total contact zone. Histogram (b) shows the total contact areas, expressed as *∑A_ind_*, per subzone measured in square micrometers. From the data, it is almost self-evident that the grain contact distribution is heterogeneous over the subzones. The highest number of contacts in the central subzones A4 and A5 constitute 19% and 15%, respectively, of the total number of contacts. On the other hand, peripheral subzones A1 and A10 bear a very low number of contacts, being 5% and 2%, respectively, of the total. This means that the core area of the contact zone is subjected to the highest activity during the whole process of microfinishing. The gradual reduction in contact numbers while approaching the edges of the contact zone indicates that the interaction of pressure with abrasive particles is also reduced in these zones. The sum of contact areas, represented by histogram (b), is equal to those values, represented by histogram (a). The total contact area in subzone A5 is the largest, followed by A4. It further proves that during the interaction of abrasive grains with workpiece material, a major load is carried out through the central subzones. On the other extreme, A1 and A10 represent the smallest total contact areas again, which indicates a very small number of interactions happening through those particular regions. The most obvious involvement of the abrasive grains in the main subzones, particularly A3 to A6, would make these zones most prominent for effective material removal. The results bring out the fact that the microfinishing process is focused in the middle of the contact zone. The highest contact numbers and total contact areas are believed to be the most important areas regarding the workpiece–abrasive film interaction in subzones A4 and A5. That is most likely due to the uneven pressure distribution applied by the roller or to the particular design of the abrasive tool, which forces the central zone. A decrease in activity can be explained for subzones A1, A2, A9, and A10 by a decrease in pressure or simply by less involvement of the abrasive particles. Results pinpoint the areas of increasing functionality of the abrasive films in the central region, as this is the area of the highest importance in terms of efficient material removal. The distribution of contact points and total contact areas can be analyzed, which will bring important information for enhancement in the design of tools and optimization of process parameters to further improve the reliability and effectiveness of the microfinishing process.

**Figure 13:**
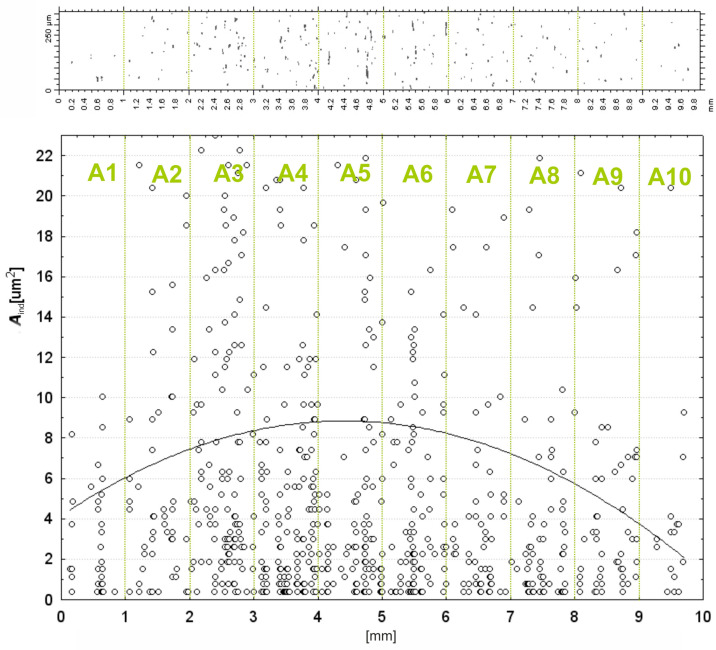
Surface areas of grain impressions *A_ind_* for individual contact zones A1...A10.

The figure depicts the meshing of discrete contact patches *A_ind_* between grinding particles and the workpiece in 10 predefined subzones of the contact zone, labeled from A1 to A10, each 1 mm in width (Figure 13). The *x*-axis is the spatial distribution of the subzones; the *y*-axis shows the measured contact areas in square micrometers, *A_ind_* [µm^2^]. Scattered data points over the graph are observed contact areas for each abrasive grain in a certain subzone. Also, in the fitted curve, an overall view of the distribution of contact area in the contact region is given; the largest single contact areas are concentrated in the middle subzones—more precisely, A4 and A5, at which the contact areas reach their maximum values.

**Figure 14:**
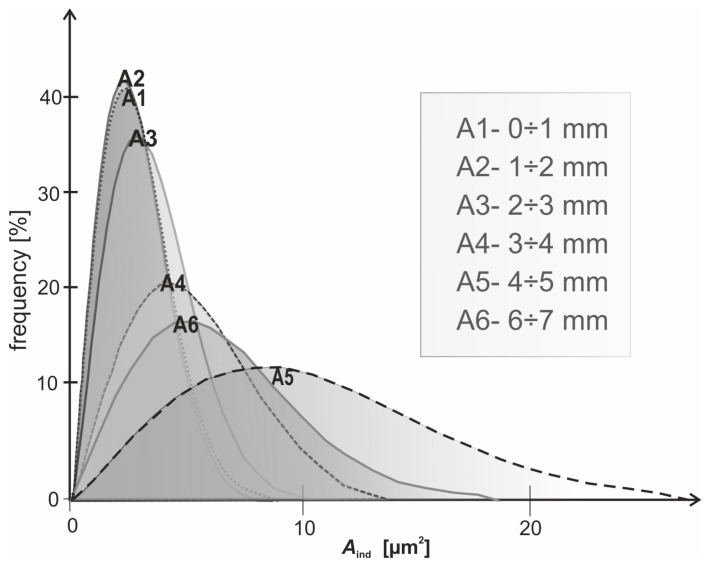
Distribution of grain contact areas across individual surface zones A1 ÷ A6.

The fitted curve is a parabolic distribution with the peak values collected in these subzones, meaning the center area in the contact zone is suffering from the most severe interaction between abrasive grains and the workpiece. Those contact zones found in the outer subzones, for example, A1, A2, A9, and A10, are felt to be much smaller, as is reflected by the lower density of points along the downward trajectory of the fitted curve. This type of observation is in agreement with the statement of reduced interaction toward the outer periphery of the contact zone. Within the subzones, there are striking variations in contact areas of individual grains, in which some grains show less contact and others equally large contact areas. Such variation originates from the random distribution and orientation of the abrasive grains on the film surface. The results point out that subzones A4, A5, and A6 are controlling as the abrasive grains make maximum contact with the workpiece (Figure 14). This central area concentration can be traced to the fact that the roller applies a continuous pressure, which is, in general, higher at the center of the contact region. The above finding agrees with the mechanical setup of microfinishing processes where the central area is designed to carry much of the operation load as a means to effectively remove materials. On the other hand, a number of factors may allude to the lower contact areas reported in peripheral subregions: reduced pressure at the outer edge of the roller and less frequent contact of the abrasive particles at the peripheral regions. This would mean that the edges of the abrasive layer are less effective in material removal and, hence, likely to have implications on the overall uniformity of the finishing operation. The variation observed for individual contact areas within all subzones is indicative of the randomness in the distribution of abrasive grains and their subsequent interactions with the workpiece. It is evident from the peak values that the central subzones are mainly controlled by larger grains, whereas smaller or less engaged grains prevail in peripheral subzones. The results underline the importance of the central contact zone (A3–A6) for the microfinishing operations since the largest contact areas occur within this zone and, hence, it becomes dominating for effective material removal. Such a reduction of contact area near the peripheries of A1, A2, A9, and A10 further emphasizes the point of optimization in abrasive film configuration and pressure distribution to improve uniformity across the whole contact zone. These results are very helpful in enhancing the performances of the abrasive films and for optimization of the microfinishing process parameters.

**Figure 15:**
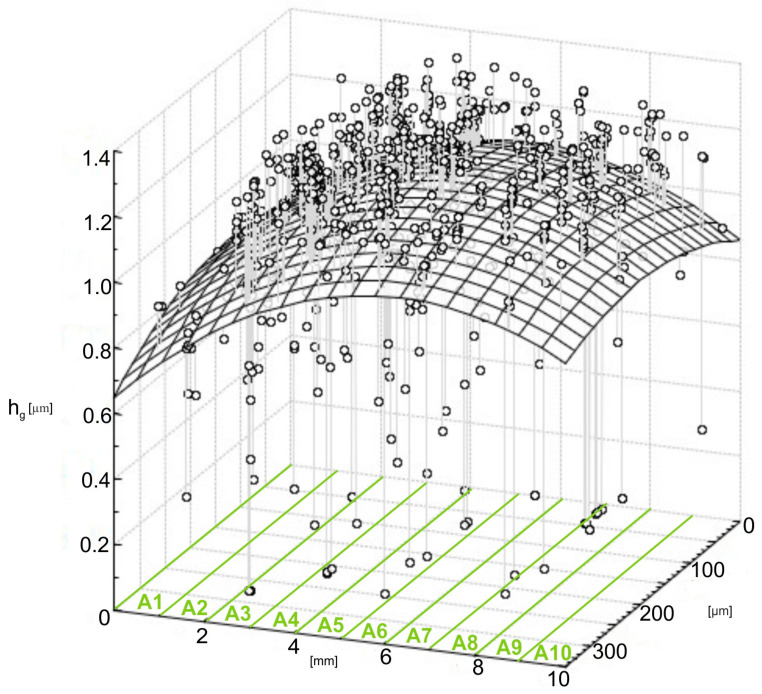
Values of abrasive film grain indentations *h_g_* in the machined material.

The size of individual abrasive grains is related to the following research penetrating the workpiece material (h_g_—penetration depth), as is shown in Figure 15. The 3D graph shows the spatial grain penetration depth distribution through the 10 predefined subzones of the contact area between A1 and A10, each 1 mm wide. The *x*-axis indicates the subzones; the *y*-axis gives the spatial distribution of grains in micrometers. The *z*-axis represents the depth of penetration in μm and *h_g_* in micrometers. The scattered data points in Figure 15 represent measured penetration depths for each individual abrasive grain located in one of the subzones, while the fitted surface provides a general view of how the penetration depths change within the contact zone. Following a similar procedure to that for analyzing the contact area *A_ind_*, the deepest grain penetrations are observed in the central subzones, mainly in A4 and A5, where the modified surface reaches the highest height. This clearly confirms that in the middle region of the contact zone, the most severe interactions between abrasive grains and the workpiece material take place. In contrast, the outer subzones (A1, A2, A9, and A10) exhibit a much smaller depth of grain penetration supported by the lower fitted surface and density of data points. This confirms previous work in the contact zones, where it was found that outer zones were less interactive due to either reduced applied pressure or reduced engagement of abrasive grains.

Further evidence for the hypothesis that the central regions bear the major share of the microfinishing load is provided by the parabolic shape of the fitted surface. The difference in penetration depths is large for each subzone. It reflects not only the stochastic nature of grain placement and orientation on the abrasive film but also the differences in the size of each grain. Larger grains will tend to penetrate deeper into the workpiece, particularly in the central regions. That is well supported by the cluster of data points in that area around the peak of the fitted surface. On the other hand, smaller or less engaged grains exhibit shallower penetration depths, mostly within peripheral subregions. Results strongly suggest that the central contact zone (A3–A6) plays a critical role in the microfinishing process, as these zones show the deepest grain penetrations and, thus, contribute the most to material removal. The necessity for further optimization in abrasive film design and pressure application for better uniformity of the entire contact zone is underlined by reduced penetration depths in peripheral zones. This assessment, in conjunction with previous findings on contact areas, *A_ind_*, will bring a deep understanding of how abrasive grains interact with the workpiece and impart much practical value in improving the performance of the abrasive film to increase efficiency in the microfinishing process.

**Figure 16:**
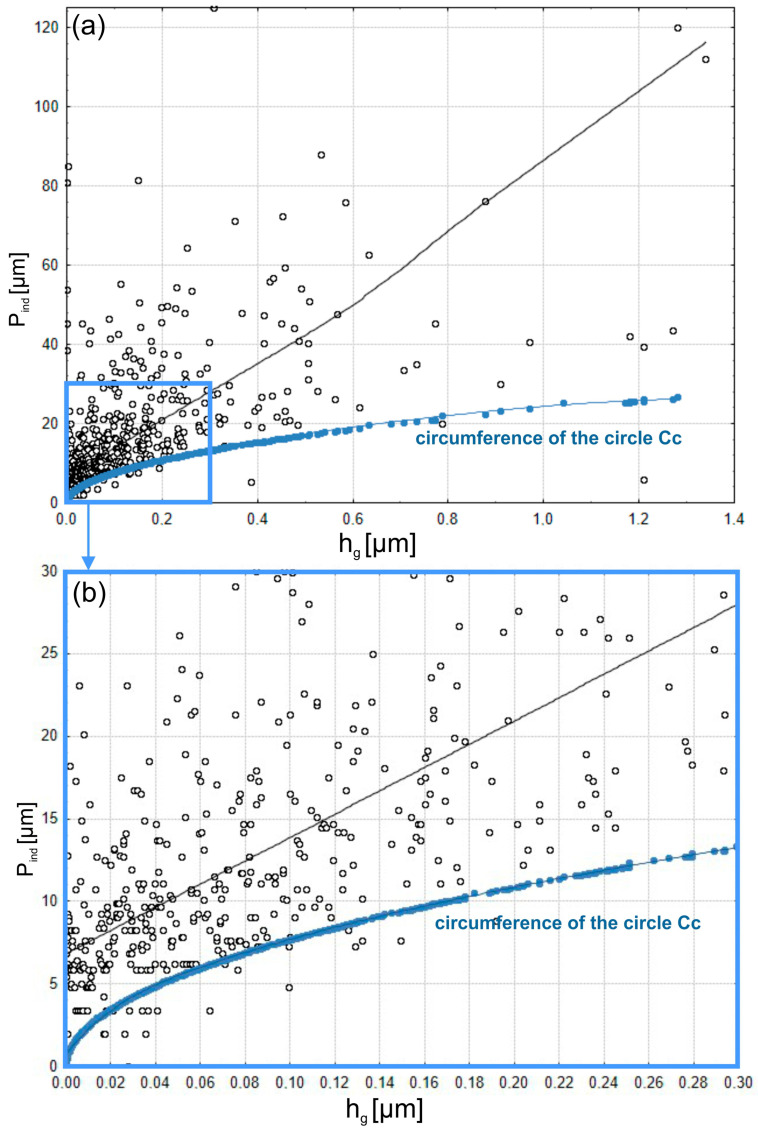
Perimeters of contact areas of abrasive grains from the 15MFF film *P_ind_* and spherical grains *Cc* with the workpiece (**a**); magnification of the smallest perimeter areas (**b**).

Figure 16 presents the results of the examination of grain-imprint circumferences (*P_ind_*) as a function of depth of penetration (*h_g_*). The upper figure shows the entire range of grain penetration depths. The lower figure enlarges only the shallowest impressions to show more clearly what is happening during the initial stages of contact. Also shown are circular projections (*Cc*), which are shown as a reference baseline. Those theoretical values correspond to the minimum value of imprint circumferences for abrasive grains of isometric shapes. From the data, it is rather obvious that, with regard to the majority of measured penetration depths, the real imprints have greater circumferences than the spherical reference model. This can clearly be seen in the deeper penetration range, where the values of *P_ind_* strongly increase and diverge even more from the theoretical *Cc* baseline. This would find an explanation in that the shapes of abrasive grains are irregular and far from the spherical approximation.

The complex geometry of grains provides more contact points and increases the perimeter of imprints with the penetration depth increase. In the shallow penetration range, shown in the magnified bottom plot, actual circumferences of imprints are closer to the theoretical value *Cc* curve. This parallelism suggests that at the earlier stages of penetration, the area of contact between the grains and the workpiece is more or less localized, and the shape of the grain apex is predominant. Still, even at these shallow depths, slight deviations can be seen, which reflect the irregularities in grain shape and orientation. The fitted trend line in the top plot confirms a non-linear relationship between the penetration depth (*h_g_*) and imprint circumference (*P_ind_*). The rapid growth of *P_ind_* at higher depths of penetration suggests that the interaction mechanism of the abrasive grains with the workpiece material is rather complex. The deviation of the *Cc* curve reflects the grain geometry effect and material deformation in the microfinishing process.

The results emphasize the significance of grain geometry in the microfinishing process. While spherical approximation gives a good first step in analyzing grain interactions, actual abrasive film performance falls under the regime of irregular grain shapes and distributions. Observed trends underline the need for further optimization in the design of abrasive films with respect to controlling grain shape and distribution for effective material removal and efficient surface finishing. The given analysis, together with previous results on contact areas (*A_ind_*) and penetration depths (*h_g_*), gives a comprehensive understanding of how the abrasive grains interact with the workpiece. The way abrasive grains contact the workpiece has been thoroughly understood. Herein, this role of grain geometry in defining contact characteristics and material removal efficiency is represented, which allows practical insight into how the performance of the abrasive film can be improved in microfinishing operations.

**Figure 17:**
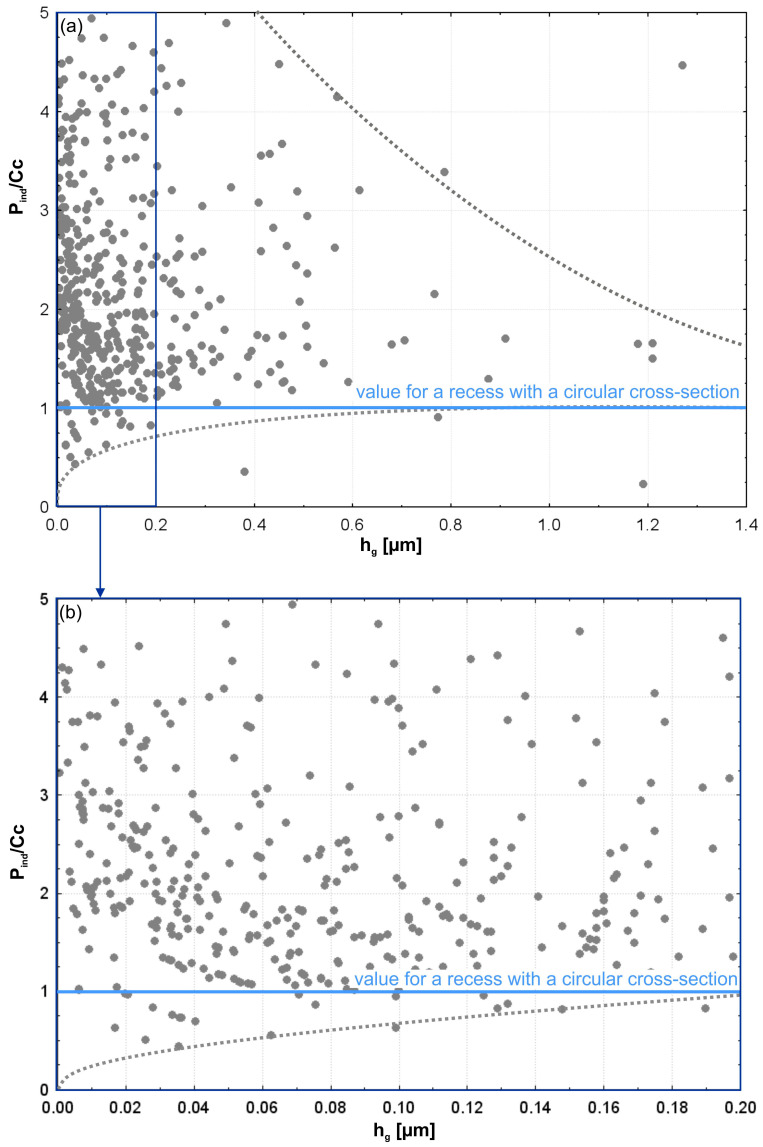
(**a**) The ratio of the perimeter of the indentation area from the abrasive grain of the MFF15 film *P_ind_* to the perimeter of the indentation from spherical grains *Cc*, illustrating the degree of surface development of the indentation (**a**); magnified view for small values (**b**).

To quantify the amount of surface development within the grain indentations, the following index was calculated: the ratio of the base circumference of the abrasive grain’s indentation (*P_ind_*) to the circumference of a spherical apex’s indentation (*Cc*). Figure 17 illustrates how this index varies with the penetration depth (*h_g_*). Upper in this viewgraph, the whole scale of penetration depths is computed. The lower one allows focusing on shallow depths, i.e., at large depths, to give insight into details crucial for initial contact situations. Results indicate that the degree of surface development decreases with increasing depth of penetration. At shallow depths, where the workpiece is predominantly interacted with by the apex of the grain, the index *P_ind_* over *Cc* is much larger than 1. Such evidence shows that the surface geometry of the apex of the grain is much more complex compared to the lower parts of the grain. For larger penetration depths, the value of *P_ind_* over *Cc* tends to be closer to 1, and hence, the spherical approximation becomes valid. That would mean the deeper parts of the grain behave more like the idealized sphere—having less obvious surface features. These findings compare with the results from Figure 16 in order to prove that the irregular geometry of abrasive grains dramatically affects the contact characteristics at shallow penetration depths. The fact that surface development is quite intensive at shallow depths suggests that material removal should belong to a dominant role played by apex geometry. As the grain goes deeper, this behavior decays in some sense: the contact surface becomes simpler and less irregular. The lower part of Figure 17 zoomed in illustrates the variability of surface development at shallow penetration depths. The scattered data points reveal big differences in grain geometry even for small depths, reflecting once again the stochastic nature of abrasive grain shapes and their interaction with the workpiece. The observed deviations from the theoretical value of 1 for *Cc* suggest that even at the shallower depths, surface irregularities of grain apexes have a primary role in the definition of the contact area and material removal efficiency. The fact that *P_ind_* decreases with an increase in *Cc* and an increase in depth means that the grain apex is very important at the early stages of microfinishing. These observations confirm the previous studies, which were carried out by some researchers on the depths of penetration and contact areas, emphasizing the very important role played by apex geometry in defining the interaction mechanism. This points out the fact that the change into simpler geometries with increasing depth hints at the reduction of contribution from the apex roughness and that the deeper parts of the grain serve mainly to stabilize the contact rather than actively removing material.

All these results underline the necessity of further optimization of abrasive grain geometry in order to improve performance in the microfinishing process. It is an index to the degree of surface development that helps explain how the grain shape influences contact mechanics and material removal. This means large improvements in efficiency and uniformity are possible by the optimization of grain geometry to take better advantage of apex interactions at shallow depths.

**Figure 18:**
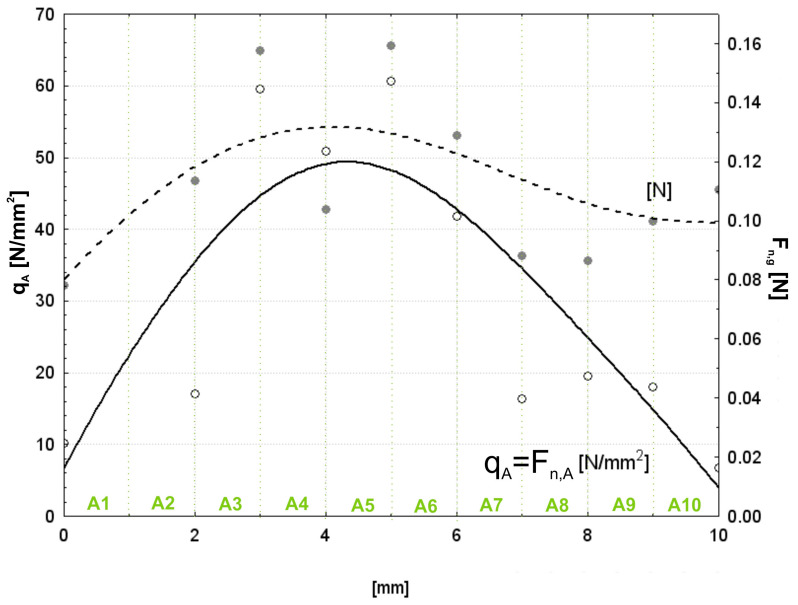
The value of the unit force and the force per single grain depending on the position within the contact zone.

The last portion of the computations was the analysis of the total contact area in a specified zone and the normal force induced by a single abrasive grain. These calculations made it possible to estimate the spread of contact characteristics and forces over ten preselected zones of contact, numbered from A1 to A10. The outcome presents the complete picture of the spatial distribution of the contact area and forces inside the contact zone. Thus, the total contact area within each zone was obtained by summation of the contact area corresponding to each interacting abrasive grain within that particular zone. Then, the normal force per grain is determined bydividing the total normal force in the zone by the number of grains in contact. It definitely brings a detailed view of how the interaction mechanics work when abrasive grains and work material interact. The calculated results for all ten zones, from A1 to A10, are listed in Table 1 and visualized in Figure 18. These results provide a basis for understanding the spatial variation of contact areas and forces, therefore pointing out the critical zones where the abrasive grains contribute most significantly to the microfinishing process.

Figure 18 illustrates the distribution of unit pressure (*q_A_*) and normal force per grain (*F_n,g_*) along the 10 pre-defined contact zones, A1 to A10, each of width 1 mm. The solid line represents the unit pressure (*q_A_*), equivalent to the unit force (*F_n,A_*) per unit area, expressed in [N/mm^2^]. The dashed line corresponds to the normal force per grain (*F_n,g_*), expressed in [N]. From the plots, one can find that there is a parabolic distribution for the unit pressure *q_A_*, and the maximum value is in the central zones A4 and A5. This may be a sign that the abrasive film and workpiece in the middle contact zones are subjected to the most intensive interaction; however, moving on to the peripheral zones A1, A2, A9, and A10, the unit pressure decreases to a great extent, reflecting the reduction in contact activity and applied pressure. The normal force per grain, *F_n,g_*, also goes in the same way, while its maximum values are again located in the regions for maximum unit pressure. Indeed, it is quite logical because in central areas, in view of sharing the greatest part of the normal load by the abrasive grains, they contribute the most to the material removal process. On the opposite, the lower force per grain in outer zones means less effective material removal at reduced interaction intensities. These results confirm how important the central contact zones, A3 to A6, are in contributing to the microfinishing process, as they provide the major part of the effective material removal. Their decreasing trends toward the edges underline the need for a process optimization approach so that there will be uniformity in pressure and force over the whole contact zone for better homogeneity and efficiency in the operation of microfinishing.

## 4. Summary and Conclusions

The objective of the study was to analyze the interaction of abrasive grains and workpieces in microfinishing operations. A few critical parameters were taken into consideration, such as unit force, unit pressure, and force applied by a single grain. It is revealed that the areas nearer to the central microfinishing zone are mainly responsible for effective material removal since they bear the highest unit pressure and forces per grain. The analysis of the contact zones and the depths of penetration proved that the regions nearest to the center bore the majority of the load during the microfinishing operation. The non-uniformity in load distribution, represented by a parabolic nature of forces and pressures, resulted in reduced material removal efficiency, as areas closer to the periphery of the contact area showed considerably smaller values. It was found to be so that contact mechanics is strongly influenced by the non-uniform geometries of abrasive particles, especially for shallow penetration depths where the apex configurations of the particles governed the interaction. This means that it is a very critical factor in the formulation of abrasive films. Moreover, from the result obtained, it could also be drawn that the peripheral areas of the contact zone underwent lower intensities of interaction; hence, there is a need for process optimization to adequately distribute the force over the whole contact area. It was proven in the study that abrasive grain geometry and pressure application could considerably improve the performance of the abrasive films; further, it would provide more homogeneous and effective microfinishing processes. Those insights provide valuable guidance in improving abrasive film technologies and microfinishing operations to attain better surface quality and process reliability.

- Regions around the central microfinishing region are equally important in efficient material removal as they have the most unit pressures and forces imposed per grain. The maximum penetration depth of the grains in these zones was 16 µm, and the total contact area in zone A5 was 1580.9 µm^2^, representing the largest contribution to material removal. The unit pressure (*q_A_*) in the central zones, such as A4 and A5, was 1.364 N/mm^2^ and 2.090 N/mm^2^, respectively, while the force per grain was 0.104 N and 0.159 N.
- Unit pressure and force per grain follow a parabolic distribution in which maximum values are confined mainly to the central zone, underlying the need for optimization in the load-carrying pattern.
- The irregular geometry of abrasive grains significantly affects contact mechanics, particularly for shallow penetration depths where apex geometry plays a dominant role. At shallow depths, the ratio of the indentation perimeter to the spherical reference (*P_ind_*/*Cc*) is significantly greater than 1, indicating the geometric complexity of the peaks.
- Peripheral areas of the contact zone show low engagement levels, which means grain engagement and pressure distribution are to be enhanced in the whole microfinishing zone. In these zones, the contact area was 129.7 µm^2^ and 177.7 µm^2^, respectively, representing the lowest values in the entire region.
- Improvements in abrasive film design and more accurate application of pressure can give rise to more consistent, efficient, and reliable microfinishing processes. The force distribution in zones A3 to A6 accounted for the largest contribution to material removal, suggesting the need to focus on these areas during process design.

## Figures and Tables

**Table 1:** Results of unit forces and pressures (*k* = 0.7).

Zone	*A_sum_^k^*	*n_i_*	∑*A_ind_* [µm^2^]	∑*A_ind/_n_i_* [um^2^]	*A_sum_^k^*/*n_i_*	*q_A_* = *F_n,A_* [N]	*F_n,g_* [N]
A1	132.0	129	177.7	1.38	1.023	10.0	0.078
A2	223.1	150	311.8	2.08	1.487	17.0	0.113
A3	781.8	378	1299.4	3.44	2.068	59.5	0.157
A4	667.1	489	968.6	1.98	1.364	50.8	0.104
A5	796.1	381	1580.9	4.15	2.090	60.6	0.159
A6	548.1	324	948.4	2.93	1.692	41.7	0.129
A7	215.0	186	279.5	1.50	1.156	16.4	0.088
A8	254.8	225	360.1	1.60	1.132	19.4	0.086
A9	235.4	180	319.6	1.78	1.308	17.9	0.100
A10	87.0	60	129.7	2.16	1.449	6.6	0.110
Sum	3940.4	2502	6375.6			***F_T_*** = 300.0	
***C*** = 300/3940.4 = 0.076							

## Data Availability

Data are contained within the article.
